# The demographic response of a deciduous shrub (the *Indigofera bungeana* complex, Fabaceae) to the Pleistocene climate changes in East Asia

**DOI:** 10.1038/s41598-017-00613-x

**Published:** 2017-04-06

**Authors:** Xue-Li Zhao, Xin-Fen Gao, Zhang-Ming Zhu, Yun-Dong Gao, Bo Xu

**Affiliations:** 1 CAS Key Laboratory of Mountain Ecological Restoration and Bioresource Utilization & Ecological Restoration and Biodiversity Conservation Key Laboratory of Sichuan Province, Chengdu Institute of Biology, Chinese Academy of Sciences, P. O. Box 416, Chengdu, Sichuan 610041 China; 2 College of Forestry, Southwest Forestry University, Kunming, 650224 China; 3 Institute of Ecology and Geobotany, Yunnan University, Kunming, 650091 China

## Abstract

East Asia harbors the highest level of floristic diversity among the world’s temperate regions. Despite the increase in phylogeographic studies of temperate plants in East Asia, far less attention has been paid to widely distributed deciduous shrubs that widespread across several floral regions. We sequenced two chloroplast DNA (cpDNA) fragments (*ndh*J-*trn*F and *trn*D-*trn*T) and one nuclear DNA (*Pgk1*) of 472 individuals from 51 populations of such a group, the *Indigofera bungeana* complex. We used population genetic data as well as ecological niche modelling to examine the evolutionary history and glacial refugia during the Last Glacial Maximum (LGM) of this group. We recovered 133 cpDNA and 68 nuclear haplotypes. The star-phylogeny of the recovered cpDNA and nuclear haplotypes and demographic analyses suggested distinct range expansion of *I. bungeana* complex have occurred during the early and middle Pleistocene. The climate change of the LGM might have affected little on the distribution of this complex based on the niche modelling. However, these climate changes and geographic isolation probably resulted in fixtures of the private haplotypes and genetic differentiations between regions. Our results suggested that this arid-tolerant species complex may have different responses to the Quaternary climate changes with those climate-sensitive species.

## Introduction

It is now well appreciated that climate oscillations during the Quaternary have profoundly shaped the geographic distributions and current genetic diversity of many temperate species in the Northern Hemisphere^1^. Two general hypotheses on forest responses to the Quaternary climate changes in East Asia have been proposed^2, 3^. Palaeovegetation data from East Asia showed that temperate forests in this region were considerably more restricted than today and would have retreated southward to c. 30°N during the LGM^2^. Conversely, Harrison *et al*. suggested that temperate forests in this region did not migrate on a large scale, but rather retained in local low-altitude refugia and formed discontinuous forest vegetation during glacial periods^3^. A limited number of phylogeographic studies appear to support the latter hypothesis. For example, in northern and northeast China, two species of shrubs, *Ostryopsis davidiana* Decne. and *Quercus mongolica* Fischer ex Ledebour, survived in multiple glacial refugia and showed regional postglacial expansions^4, 5^ while in southern and southeast China multiple refugia were recovered for a few trees and accompanying species, such as *Tetrastigma hemsleyanum* Diels & Gilg^6^, the East Asian *Kirengeshoma* ^7^, and the species of the fir genus^8^. In subtropical China, the evergreen broad-leaved forest constituents conform to either an *in situ* survival model or an expansion-contraction model, such as *Castanopsis tibetana*, *Machilus thunbergii* and *Schima superba* ^9^, *Sargentodoxa cuneata* ^10^, and *Loropetalum chinense* ^11^. In addition, in the eastern Himalaya, some species from multiple refugia during the Last Glacial Maximum expanded their range to colonize extensive regions before the middle Quaternary^12–14^, although some climate-sensitive trees retreated and recolonized high-altitude regions after the LGM^15, 16^. Up to now, no studies have examined the phylogeographic structure of a single species/species complex or monophyletic group whose current distributions cover all of these regions in East Asia.

In this study, we focused on the phylogeographic patterns of the *Indigofera bungeana* complex (Fabaceae), a complex of deciduous shrubs widespread in temperate East Asia (between c. 23°N and 45°N in latitude, 95°E and 135°E in longitude), with a continuous geographic distribution covering southern China, northern China and the Hengduan Mountains region (HMR). Four species have been ascribed to this complex^17^, *I. bungeana* Walpers, *I. amblyantha* Craib, *I. silvestrii* Pampanini, and *I. ramulosissima* Hosokawa. Species delimitations between them remains unclear due to the lack of clear morphological and genetic gaps. Our unpublished phylogenetic analyses suggested that numerous individuals of each species intermixed, but formed a highly supported monophyletic clade sister to the species of *Indigofera* from the Cape region of South Africa. We therefore treated them as a single evolutionary lineage in our phylogeographic analyses. Members of this complex grow in sunny, arid habitats at elevations between 100‒2700 m^17^. Their widespread distribution provides a unique opportunity to examine how plants responded to past climate changes over a large region in East Asia.

We sequenced two types of DNA fragments with contrasting backgrounds of inheritance. First, two maternally inherited chloroplast DNAs (cpDNAs) were used, as in most phylogeographic studies^16, 18^, due to the merits of rare recombinations and smaller effective population size^19^. This type of population genetic data allows an inference of historical range shifts and recolonization routes^20–22^. Second, we also sequenced one nuclear DNA fragment. Population data from nuclear genetic polymorphisms can confirm the phylogeographic inferences from cpDNA^23–26^. Sequence variation data from a single nuclear locus is becoming popular for such an aim^23, 27^.

We finally used ecological niche modelling to infer the possible distributions of this complex during the LGM in East Asia. We expected that the simulated distributions should be consistent with the phylogeographic inferences of the population genetic data. We aimed to address the following questions: (1) Are phylogeographic inferences from cpDNA data consistent with those from nuclear DNA data? (2) When and how did this complex obtain its widespread distribution in East Asia? (3) Did the *I. bungeana* complex retreat southward or survive *in situ* during the LGM?

## Results

### cpDNA variation and haplotype structure

**Figure 1:**
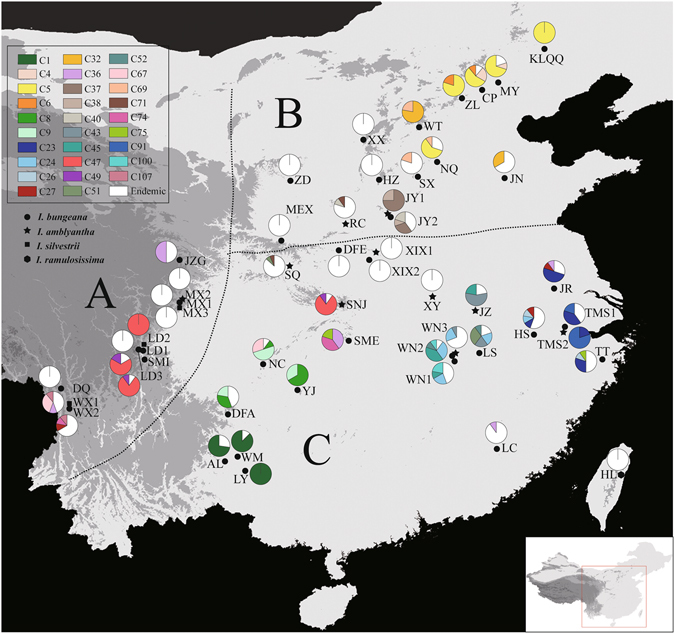
Geographic distribution of cpDNA haplotypes detected in *Indigofera bungeana* complex. The haplotypes found in more than one population are color-coded, while private haplotype particular to each population are shown in white. Figure was generated in DIVA-GIS 7.5 (http://www.diva-gis.org).

The total alignment of the *ndh*J-*trn*F and *trn*D-*trn*T fragments across the 472 individuals sampled was 2933 bp, containing 155 substitutions and 43 indels (insertion/deletion) (4‒116 bp). A total of 133 chlorotypes (C1‒C133) was identified, 104 (78.2%) of which occurred in a single population (Table 1). The most common haplotypes, C5, C23 and C24, were found in 5 (9.8%) populations, respectively. Total haplotype (*H*_d_) and nucleotide (*π*) diversity of the cpDNA data was 0.982 and 0.0033, respectively. Seven of the 51 populations surveyed contained only one haplotype, whereas the remaining populations were polymorphic (Table 1; Fig. 1).

**Table 1:** Locations of populations of *Indigofera bungeana* complex sampled, sample sizes (n), frequencies of chloroplast and *Pgk1* haplotypes per population, the geographic region (Figs 1 and 2) and lineage (Figs 3a and 4a) for each population.

Species	Population	Location	Longitude	Latitude	E	cpDNA			*Pgk1*		
	Code (Region)		(E)	(N)	(m)	n	Chlorotype nos.	Lineage	n	Haplotype nos.	Lineage
*I. amblyantha*	JY1 (B)	Jiyuan, HEN	112°16′04″	35°11′23″	1650	4	C37(3), C38(1)	C	6	H2(2), H21(2), H22(1), H23(1)	III
	JZ (C)	Jinzhai, AH	115°51′22″	31°14′10″	427	9	C43(5), C44(2), C45(2)	C	16	H6(9), H21(4), H22(1), H26(1), H27(1)	II
	MX2 (A)	Maoxian, SC	103°51′36″	31°41′24″	1800	12	C61(12)	C	22	H22(4), H43(17), H44(1)	III
	RC (B)	Ruicheng, SX	110°32′28″	34°47′33″	1350	10	C40(1), C70(4), C71(1), C72(1), C73(3)	C	20	H5(1), H6(6), H21(9), H48(1), H49(1), H50(1), H51(1)	III
	SNJ (C)	Shennongjia, HUB	110°22′58″	31°28′29″	1367	10	C67(1), C77(9)	C	20	H6(18), H21(2)	III
	SQ (C)	Shiquan, SHX	108°15′00″	33°03′09″	530	15	C5(1), C71(1), C78(3), C79(1), C80(4), C81(3), C82(1), C83(1)	C	16	H2(1), H16(2), H17(2), H21(2), H43(6), H54(1), H55(1), H56(1)	I, III
	TMS2 (C)	Tianmushan, ZJ	119°27′22″	30°20′16″	600	5	C23(1), C91(4)	C	10	H2(1), H21(2), H54(7)	III
	WN2 (C)	Wuning, JX	114°55′12″	29°18′36″	500	10	C24(3), C45(4), C52(1), C100(1), C103(1)	C	18	H16(5), H17(10), H37(1), H38(1), H61(1)	I
	WN3 (C)	Wuning, JX	114°55′12″	29°22′12″	850	13	C24(3), C43(1), C104(1), C105(8)	C	26	H6(8), H21(13), H54(1), H62(4)	III
	XIX1 (C)	Xixia, HEN	111°47′44″	33°37′38″	1500	10	C116(2), C117(2), C118(5), C119(1), C120(1)	C	20	H6(11), H21(5), H65(2), H66(2)	III
	XY (C)	Xinyang, HEN	114°04′24″	31°49′07″	438	9	C124(4), C125(1), C126(1), C127(1), C128(2)	C	16	H2(1), H6(6), H21(7), H68(2)	III
*I. bungeana*	AL (B)	Anlong, GZ	105°35′24″	25°04′48″	1415	11	C1(8), C2(3)	C	22	H1(20), H2(2)	III
	CP (B)	Changping, BJ	116°06′53″	40°13′57″	150	9	C3(1), C4(2), C5(5), C6(1)	C	20	H2(8), H3(2), H4(6), H5(2), H6(1), H7(1)	III
	DFA (C)	Dafang, GZ	105°43′48″	26°59′24″	1377	9	C7(4), C8(3), C9(2)	B, C	20	H1(20)	III
	DFE (C)	Danfeng, SHX	110°16′12″	33°42′36″	570	10	C10(2), C11(1), C12(3), C13(1), C14(1), C15(1), C16(1)	C	20	H8(1), H9(2), H10(1), H11(13), H12(1), H13(1), H14(1)	I, III
	DQ (A)	Deqin, YN	98°54′36″	28°01′48″	1930	7	C17(6), C18(1)	C	18	H1(16), H2(2)	III
	HS (C)	Huangshan, AH	118°14′23″	31°14′31″	500	9	C20(1), C21(1), C22(1), C23(1), C24(1), C25(2), C26(1), C27(1)	C	20	H15(7), H16(1), H17(3), H18(7), H19(2)	I, III
	HZ (B)	Huozhou, SX	111°55′28″	36°36′14″	1150	10	C28(1), C29(8), C30(1)	C	16	H2(11), H20(5)	III
	JN (B)	Jinan, SD	117°03′36″	36°38′24″	250	9	C31(6), C32(3)	C	20	H2(17), H3(3)	III
	JR (C)	Jurong, JS	119°05′15″	32°08′33″	150	10	C23(5), C27(1), C33(1), C34(1), C35(1), C36(1)	C	14	H15(4), H16(2), H18(6), H19(2)	I, III
	JY2 (B)	Jiyuan, HEN	112°16′04″	35°11′23″	1250	10	C37(3), C38(1), C39(1), C40(2), C41(3)	C	16	H2(10), H24(2), H25(4)	III
	JZG (A)	Jiuzhaigou, SC	103°51′01″	33°17′24″	2200	12	C36(6), C42(6)	C	16	H2(2), H3(1), H6(12), H22(1)	III
	KLQQ (B)	Kelaqinqi, IM	118°41′02″	41°55′17″	600	10	C5(10)	C	20	H2(3), H3(1), H5(4), H7(3), H22(4), H28(1), H29(1), H30(3)	III
	LC (C)	Liancheng, FJ	116°42′45″	25°35′06″	360	10	C36(1), C46(9)	C	20	H15(13), H17(1), H18(6)	I
	LD1 (A)	Luding, SC	102°10′12″	29°37′12″	1150	12	C47(8), C48(2), C49(2)	B, C	20	H31(2), H32(18)	III
	LD3 (A)	Luding, SC	102°04′48″	29°37′48″	1800	9	C50(9)	C	16	H32(16)	III
	LS (C)	Lushang, JX	116°01′12″	29°30′36″	650	10	C24(2), C43(2), C51(3), C52(1), C53(1), C54(1)	C	18	H16(2), H17(8), H35(1), H36(1), H37(4), H38(1), H39(1)	I
	LY (C)	Leye, GX	106°25′12″	24°40′48″	1000	10	C1(10)	C	22	H1(20), H2(2)	III
	MEX (B)	Meixian, SHX	107°53′44″	34°05′14″	830	9	C55(5), C56(2), C57(1), C58(1)	C	20	H3(5), H6(10), H21(1), H29(1), H40(1), H41(2)	III
	MX1 (A)	Maoxian, SC	103°43′48″	31°34′48″	1700	8	C59(7), C60(1)	C	20	H2(3), H6(14), H42(3)	III
	MY (B)	Miyun, BJ	116°47′11″	40°34′22″	440	10	C4(1), C5(7), C64(1), C65(1)	C	20	H2(13), H3(1), H4(1), H5(2), H7(2), H30(1)	III
	NC (C)	Nanchuan, CQ	107°09′36″	29°01′12″	1300	10	C8(1), C9(5), C66(1), C67(3)	B, C	12	H16(8), H38(1), H47(3)	I
	NQ (B)	Neiqiu, HEB	114°16′48″	37°18′36″	500	10	C5(6), C68(3), C69(1)	C	20	H2(15), H4(1), H22(4)	III
	SME (C)	Shimen, HUN	110°51′36″	29°58′12″	335	5	C36(2), C74(2), C75(1)	C	6	H16(1), H38(1), H47(2), H52(2)	I, III
	SMI (A)	Shimian, SC	102°18′36″	29°15′36″	850	10	C47(8), C49(1), C76(1)	B, C	20	H32(17), H33(2), H53(1)	III
	SX (B)	Shexian, HEB	113°30′13″	36°43′45″	608	10	C69(2), C84(1), C85(2), C86(1), C87(1), C88(2), C89(1)	C	20	H2(9), H3(2), H22(3), H57(4), H58(2)	III
	TMS1 (C)	Tianmushan, ZJ	119°27′22″	30°20′16″	450	5	C23(2), C90(1), C91(1), C92(1)	C	10	H15(6), H18(4)	I
	TT (C)	Tiantai, ZJ	121°02′32″	29°14′28″	753	10	C23(3), C26(1), C75(1), C93(1), C94(1), C95(1), C96(1), C97(1)	C	14	H1(1), H2(1), H15(5), H18(5), H59(2)	I, III
	WM (C)	Wangmo, GZ	106°06′36″	25°19′48″	1030	8	C1(7), C98(1)	C	22	H1(22)	III
	WN1 (C)	Wuning, JX	115°00′36″	29°10′12″	150	10	C24(2), C45(1), C99(1), C100(2), C101(1), C102(2)	C	20	H16(2), H17(10), H37(2), H38(1), H39(1), H60(4)	I
	WT (B)	Wutai, HEB	113°32′10″	38°43′09″	1100	9	C32(7), C69(2)	C	14	H2(14)	III
	WX2 (A)	Weixi, YN	99°16′12″	27°12′36″	2320	9	C27(1), C74(1), C107(1), C111(2), C112(1), C113(1), C114(1), C115(1)	C	12	H16(3), H17(4), H38(1), H47(2), H60(1), H64(1)	I
	XIX2 (C)	Xixia, HEN	111°29′43″	33°18′42″	650	10	C121(10)	C	16	H1(5), H16(2), H67(9)	I, III
	XX (B)	Xingxian, SX	111°16′37″	38°13′08″	1633	9	C122(7), C123(2)	C	18	H2(17), H22(1)	III
	YJ (C)	Yinjiang, GZ	108°33′36″	27°59′24″	765	9	C8(6), C9(3)	B	10	H1(8), H17(2)	I, III
	ZD (B)	Zhidan, SHX	108°16′35″	36°33′18″	1220	9	C129(3), C130(4), C131(2)	C	20	H3(1), H5(5), H6(7), H20(5), H22(1), H29(1)	III
	ZL (B)	Zhuolu, HEB	115°17′26″	39°53′45″	616	10	C5(4), C6(1), C132(4), C133(1)	C	14	H2(2), H5(8), H6(1), H22(3)	III
*I. ramulosissima*	HL (C)	Hualian, TW	121°33′36″	24°10′48″	650	1	C19(1)	C	2	H15(2)	I
*I. silvestrii*	LD2 (A)	Luding, SC	102°17′24″	29°51′01″	2280	9	C47(9)	B	16	H32(9), H33(5), H34(2)	III
	MX3 (A)	Maoxian, SC	103°43′48″	31°34′48″	1435	9	C62(7), C63(2)	A	20	H45(16), H46(4)	III
	WX1 (A)	Weixi, YN	99°16′12″	27°12′36″	2320	9	C36(1), C67(3), C106(1), C107(1), C108(1), C109(1), C110(1)	C	16	H1(1), H16(8), H17(3), H38(2), H60(1), H63(1)	I, III

Frequencies of chlorotypes and *Pgk1* haplotypes are shown in parentheses. Private haplotype particular to each population are underlined.

Abbreviations: HEN, Henan; SC, Sichuan; HUB, Hubei; SX, Shanxi; SHX, Shannxi; ZJ, Zhejiang; JX, Jiangxi; GZ, Guizhou; BJ, Beijing; YN, Yunnan; AH, Anhui; SD, Shandong; JS, Jiangsu; IM, Inner Mongolia; FJ, Fujian; GX, Guangxi; CQ, Chongqing; HEB, Hebei; HUN, Hunan; Taiwan, TW.

**Figure 2:**
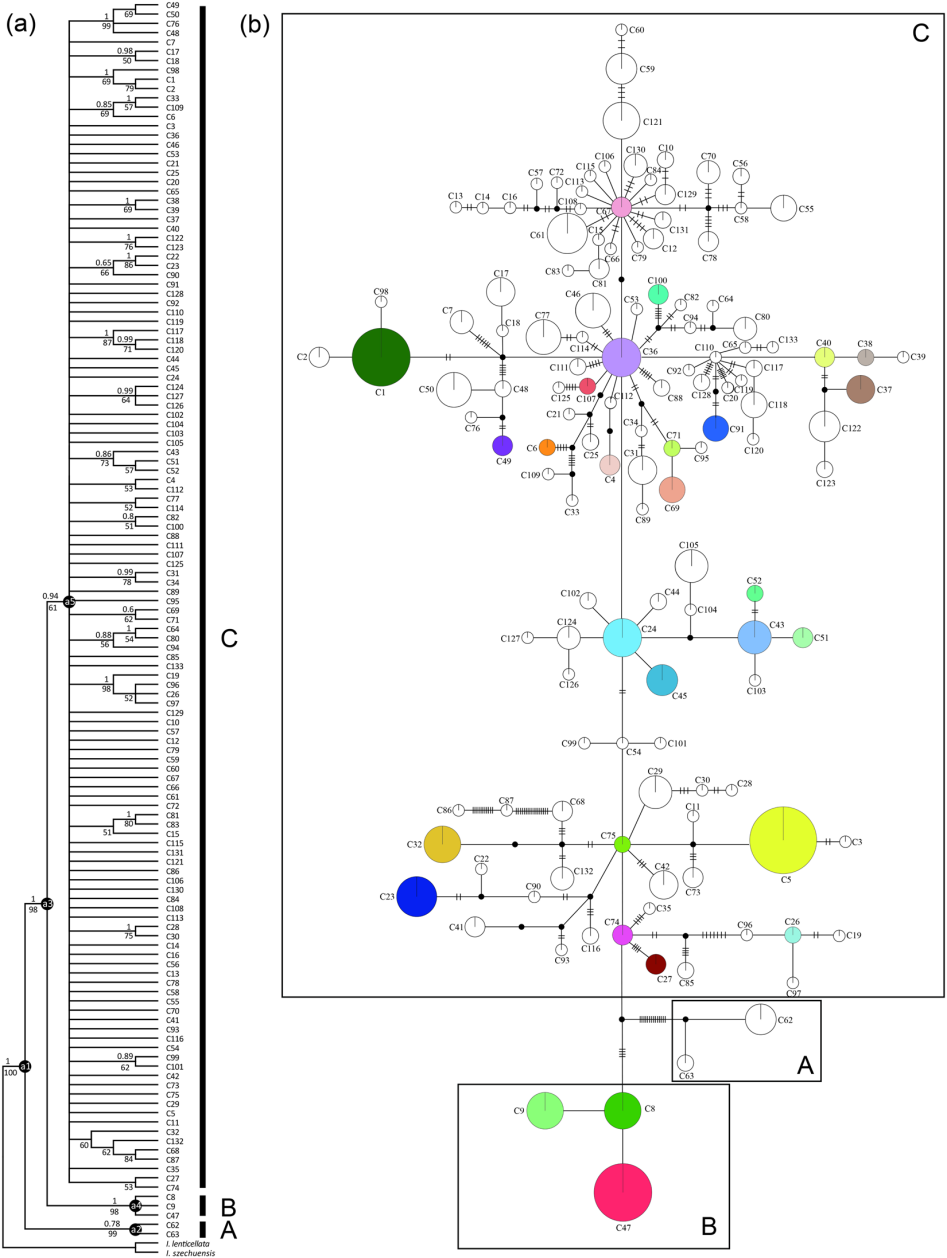
The evolutionary relationships among cpDNA haplotypes of *Indigofera bungeana* complex. (**a**) NJ phylogenetic tree of the 133 cpDNA haplotypes. Numbers above/below branches represent Bayesian posterior probabilities/NJ support values. (**b**) Maximun parsimony network. The size of circles corresponds to the frequency of each haplotype and black dots represent missing haplotypes (not sampled or extincted). Lineages (A, B, C) and clades (a1, a2, a3, a4, a5) correspond to the lineages and clades in Table 1 and Supplementary Table S1.

Phylogenetic trees reconstructed using NJ and Bayesian methods were largely consistent in topology. All chlorotypes from the *I. bungeana* complex comprised a monophyletic lineage with three clades (Fig. 2). The basal A clade included only two chlorotypes, C62 and C63, which were restricted to a single population of *I. silvestrii* (HMR: MX3); the second B clade contained three, C8, C9 and C47, distributed in the Hengduan Mountains region and southern China. Clade C included all the remaining numerous chlorotypes with unresolved relationships, indicating radiative diversification. The haplotype network showed the star phylogeny, and most cpDNA haplotypes were arranged as a radiative phylogenetic tree relative to the central ones, e.g. C67, C36, C24, C75, C74 and C8 (Fig. 1). The dating analyses under different substitution rates suggested that the common ancestor of the *I. bungeana* complex originated during the Pliocene, and those of clades A, B and C in the Pliocene to early Pleistocene. Diversification of haplotypes in clade C, which comprised almost all the chlorotypes, was estimated to have occurred before the LGM (see Supplementary Table S1).

### *Pgk1* variation and haplotype structure

**Figure 3:**
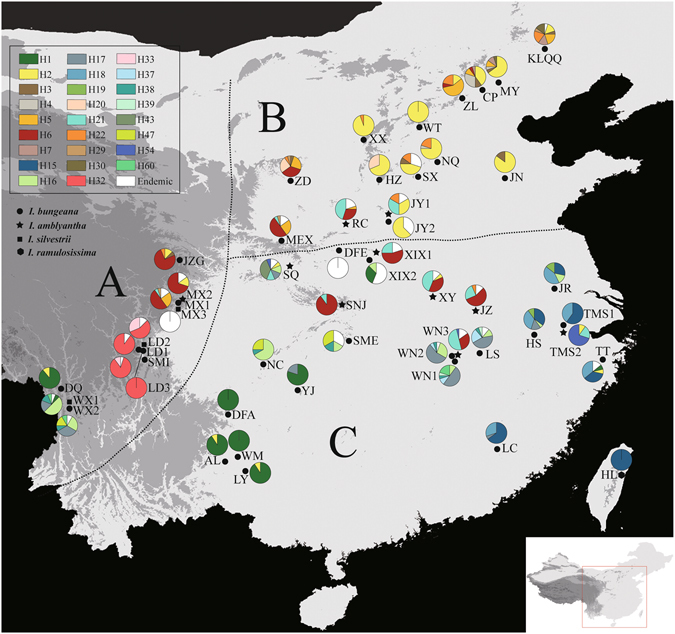
Geographic distribution of *Pgk1* haplotypes detected in *Indigofera bungeana* complex. The haplotypes found in more than one population are color-coded, while private haplotype particular to each population are shown in white. Figure was generated in DIVA-GIS 7.5 (http://www.diva-gis.org.).

The alignment of *Pgk1* across the 434 individuals was 792 bp, containing 58 substitutions and 8 indels (4‒28 bp). These polymorphisms defined 68 haplotypes, with 42 (61.8%) haplotypes unique to a single population (Table 1). The most common haplotypes H2 and H6 occurred in 20 (39.2%) and 13 (25.7%) populations, respectively. Total haplotype (*H*_d_) and nucleotide (*π*) diversity of the *Pgk1* was 0.927 and 0.0106, respectively. Among the 51 populations surveyed, five were fixed for a single haplotype, and the remaining ones were polymorphic (Table 1; Fig. 3).

**Figure 4:**
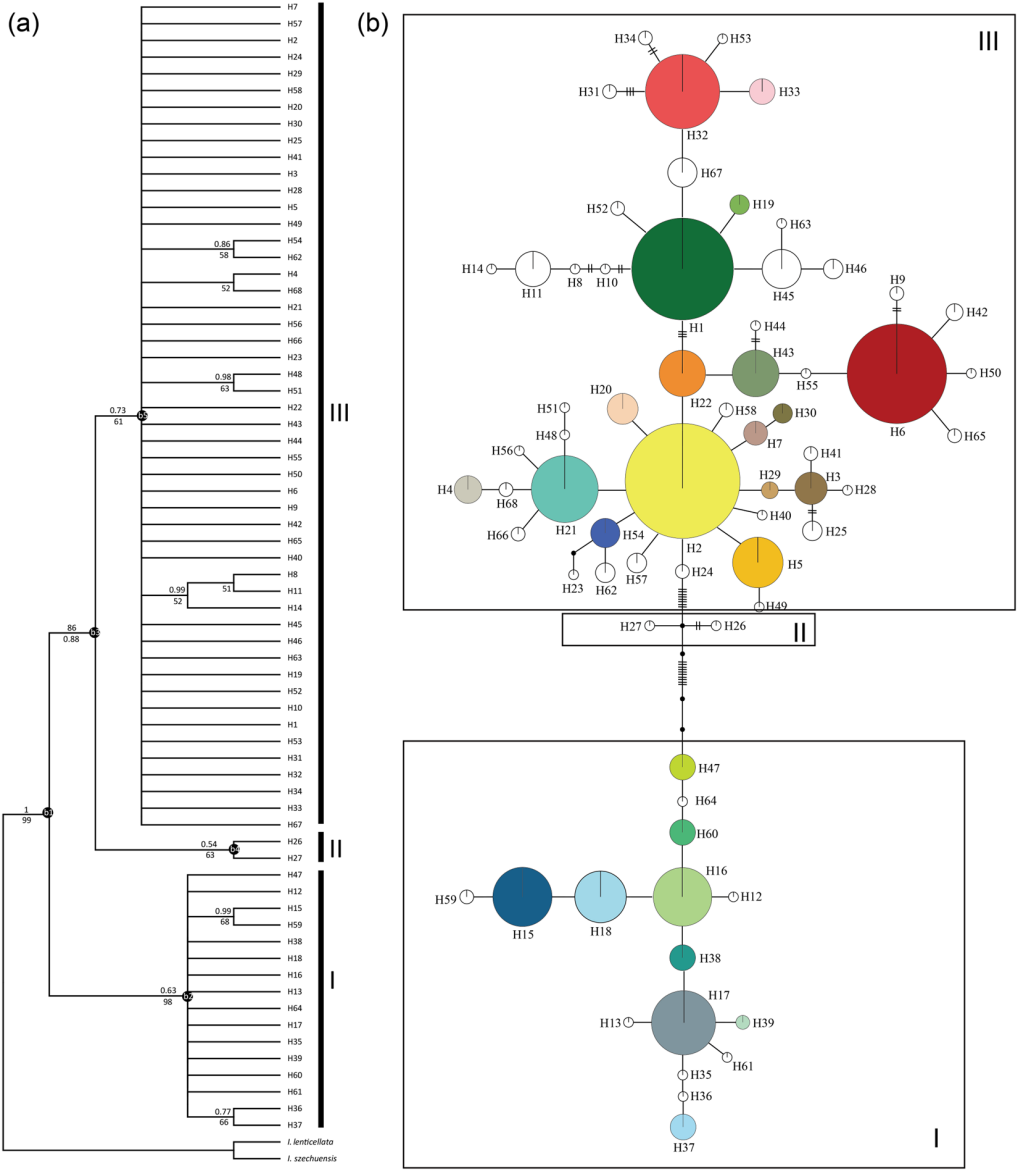
The evolutionary relationships among *Pgk1* haplotypes of *Indigofera bungeana* complex. (**a**) NJ phylogenetic tree of the 68 *Pgk1* haplotypes. Numbers above/below branches represent Bayesian posterior probabilities/NJ support values. (**b**) Maximun parsimony network. The size of circles corresponds to the frequency of each haplotype and black dots represent missing haplotypes (not sampled or extincted). Lineages (I, II, III) and clades (b1, b2, b3, b4, b5) correspond to the lineages and clades in Table 1 and Supplementary Table S1.

Nuclear (*Pgk1*) haplotypes clustered into three clades (Fig. 4). The basal clade comprised the haplotypes occurring in the HMR and southern China, and clade II included H26 and H27 that were restricted to a population of *I. amblyantha* (southern China: JZ), while the clade III comprised the remaining haplotypes that occurred throughout the whole distribution areas of *I. bungeana* complex. The haplotype network showed the same star phylogeny with most haplotypes relative to the central ones (e.g. H1, H2, H16 and H17) which occurred at a high frequency (Fig. 4b). The crown age of the *I. bungeana* complex estimated from *Pgk1* was 1.47 (95% HPD: 0.75‒2.37) Ma. The common ancestors of clades I, II and III were estimated to have occurred 0.67 (95% HPD: 0.26‒1.28) Ma, 0.35 (95% HPD: 0.01‒0.93) Ma and 0.98 (95% HPD: 0.51‒1.66) Ma (Table S3), respectively.

### Genetic differentiation

The SAMOVA analysis failed to uncover any reliable population genetic group in either the cpDNA or nuclear datasets (see Supplementary Fig. S1). We therefore divided all the populations into three groups according to the classical phytogeographic boundaries defined by Wu & Wu^28^: (A) Hengduan Mountains region (HMR), (B) Northern China, (C) Southern China (see Figs 1 and 2).

The level of total genetic diversity *H*_T_ (cpDNA: 0.991; *Pgk1*: 0.939) across the overall populations was much higher than the average within-population gene diversity *H*_S_ (Table 2). The highest genetic diversity occurred in southern China (cpDNA: 0.982; *Pgk1*: 0.922), in accordance with the occurrence of the most divergent haplotypes in this region (Figs 1 and 2). For both cpDNA and *Pgk1* datasets, a significantly larger *N*_ST_ than *G*_ST_ value across overall populations was detected (Table 2), indicating the presence of a significant phylogeographic structure.

**Table 2:** Estimates of average gene diversity within populations (*H*_S_) of *Indigofera bungeana* complex, total gene diversity (*H*_T_), inter-population (*G*_ST_), and number of substitution types (*N*_ST_) for cpDNA and *Pgk1* across regions.

Region	cpDNA					*Pgk1*				
	n	*H*_S_	*H*_T_	*G*_ST_	*N*_ST_	*n*	*H*_S_	*H*_T_	*G*_ST_	*N*_ST_
Hengduan Mountains region	27	0.389	0.962	0.595	0.792**	22	0.391	0.909	0.570	0.871**
Northern China	39	0.581	0.954	0.392	0.494	23	0.608	0.814	0.253	0.260
Southern China	72	0.626	0.982	0.362	0.525**	39	0.514	0.922	0.442	0.761**
Total	133	0.549	0.991	0.446	0.578**	69	0.510	0.939	0.457	0.800**

n, no. of haplotypes. **P < 0.001.

Hierarchical AMOVA revealed low levels of regional and species differentiation (Table 3; see Supplementary Figs S2–S5). Variations among regions accounted for 5.26% and 23.53% of the total genetic variation for cpDNA and *Pgk1* datasets, respectively. Populations in the HMR and southern China showed significantly higher variation among populations than within populations, while extremely lower differentiation among populations than within populations in northern China (Table 3). The highest level of genetic differentiation among populations (cpDNA: PV = 77.54%, *F*_ST_ = 0.775; *Pgk1*: PV = 86.74%, *F*_ST_ = 0.867) was observed in the HMR, which was consistent with the significantly higher *G*_ST_ values than in other regions (Table 3). Complex and heterogeneous climate and topography may serve as a favorable condition for isolation, drift and barriers of gene flow in the HMR. When each species was analyzed separately, differences among species explained 7.56% (*F*_CT_ = 0.076) and 4.76% (*F*_CT_ = 0.048), those among populations within species 53.33 (*F*_SC_ = 0.577) and 76.12% (*F*_SC_ = 0.799), and those within populations 39.11% (*F*_ST_ = 0.609) and 19.11% (*F*_ST_ = 0.809) of the total cpDNA and nuclear DNA genetic variation, respectively (Table 3).

**Table 3:** Hierarchical analysis of molecular variance (AMOVA) of cpDNA and *Pgk1* for *Indigofera bungeana* complex, partitioned by species and region, respectively.

Partitioning	Source of variation	cpDNA				*F*-statistics	*Pgk1*				*F*-statistics
		d.f.	SS	VC	PV (%)		d.f.	SS	VC	PV (%)	
By region	Among regions	2	135.039	0.262	5.26	*F*_CT_ = 0.053^**^	2	704.066	1.095	23.53	*F*_CT_ = 0.235^**^
	Among populations	48	1310.154	2.723	54.67	*F*_SC_ = 0.577^**^	48	2251.382	2.719	58.45	*F*_SC_ = 0.764^**^
	Within populations	421	839.881	1.995	40.06	*F*_ST_ = 0.599^**^	817	684.495	0.838	18.01	*F*_ST_ = 0.820^**^
Hengduan Mountains region	Among populations	10	464.935	4.697	77.54	*F*_ST_ = 0.775^**^	10	559.867	3.123	86.74	*F*_ST_ = 0.867^**^
	Within populations	95	129.263	1.361	22.46		185	88.322	0.477	13.26	
Northern China	Among populations	14	297.642	2.064	47.18	*F*_ST_ = 0.472^**^	14	43.593	0.144	19.43	*F*_ST_ = 0.194^**^
	Within populations	123	284.156	2.310	52.82		247	147.968	0.599	80.57	
Southern China	Among populations	24	538.577	2.238	51.59	*F*_ST_ = 0.516^**^	24	1647.923	4.136	78.03	*F*_ST_ = 0.780^**^
	Within populations	203	426.463	2.101	48.41		385	448.204	1.164	21.97	
By species	Among species	3	140.890	0.385	7.56	*F*_CT_ = 0.076^*^	3	208.638	0.209	4.76	*F*_CT_ = 0.048^**^
	Among populations	47	1295.303	2.720	53.33	*F*_SC_ = 0.577^**^	47	2746.810	3.337	76.12	*F*_SC_ = 0.799^**^
	Within populations	421	839.881	1.995	39.11	*F*_ST_ = 0.609^**^	817	684.495	0.838	19.11	*F*_ST_ = 0.809^**^
*I. amblyantha*	Among populations	10	220.881	2.096	52.50	*F*_ST_ = 0.525^**^	10	356.539	2.021	65.39	*F*_ST_ = 0.654^**^
	Within populations	96	182.073	1.897	47.50		179	191.461	1.070	34.61	
*I. bungeana*	Among populations	35	921.163	2.587	55.02	*F*_ST_ = 0.550^**^	35	2235.144	3.645	82.65	*F*_ST_ = 0.826^**^
	Within populations	301	636.697	2.115	44.98		588	450.084	0.765	17.35	
*I. silvestrii*	Among populations	2	153.259	8.417	90.54	*F*_ST_ = 0.905^**^	2	155.127	4.451	83.55	*F*_ST_ = 0.835^**^
	Within populations	24	21.111	0.880	9.46		49	42.950	0.877	16.45	
*I. ramulosissima*	Among populations	/	/	/	/	/	/	/	/	/	/
	Within populations	/	/	/	/	/	/	/	/	/	/
Total populations	Among populations	50	1436.193	2.891	59.17	*F*_ST_ = 0.592^**^	50	2955.448	3.429	80.36	*F*_ST_ = 0.804^**^
	Within populations	421	839.881	1.995	40.83		817	684.495	0.838	19.64	

d.f., degree of freedom; SS, sum of squares; VC, variance of components; PV, percentage of variation *P < 0.01, **P < 0.001.

### Tests of demographic expansion

Under a model of population expansion, the major clades identified in the cpDNA (clade A, B and C) and *Pgk1* (clade I, II and III) phylogeny displayed a bimodal or multimodal mismatch distribution (Fig. S6). However, none (except cpDNA clade A and *Pgk1* clade I) of the statistical comparisons between these observed distributions and simulated ones under a sudden expansion model significantly rejected the expansion model (*P* values > 0.05 based on *SSD* and *H*_Rag_, Table 4). Nonsignificant *SSD* and raggedness index, as well as a significant large negative *F*_S_ (C: *F*_S_ = −24.002, *P* < 0.001; III: *F*_S_ = −21.123, *P* < 0.01) and Tajima’s *D* (C: *D* = −0.599, *P* = 0.306; III: *D* = −2.096, *P* < 0.001) values, indicated a historical demographic expansion within cpDNA clade C and *Pgk1* clade III. Based on the corresponding *τ*, and assuming minimum and maximum substitution rates of 1.0 × 10^−9^ and 3.0 × 10^−9^ s s^−1^y^−1^, the expansion of clade C was estimated to have occurred at 698 (95% CI: 493‒961) and 232 (95% CI: 164‒320) thousand years ago (Kya), respectively. Bayesian skyline plots suggested that effective population size of the cpDNA clade C increased quickly (Fig. S7). The expansion for the *Pgk1* clade III was estimated to have occurred 60 (95% CI: 5‒220) Kya (Table 4).

**Table 4:** Mismatch distribution and neutrality tests for populations of clades of *Indigofera bungeana* complex.

	Clade	Tajima’s *D* (*P*-value)	Fu’s *F*_S_ (*P*-value)	*τ* (95% CI)	*H*_Rag_ (*P*-value)	*SSD* (*P*-value)	Expansion time (Ma)
cpDNA	A	0.196 (0.513)	1.591 (0.794)	0.000 (0.000‒0.506)	0.676 (0.890)	0.302 (0.000)	NC
	B	−0.021 (0.504)	4.586 (0.961)	0.021 (0.000‒4.496)	0.053 (0.680)	0.058 (0.570)	NC
	C	−2.096 (0.000)	−24.002 (0.000)	8.185 (5.779‒11.267)	0.005 (0.400)	0.001 (0.310)	*t*_min_ = 0.698 (0.493‒0.961), *t*_max_ = 0.232 (0.164‒0.320)
*Pgk1*	I	1.345 (0.868)	−1.013 (0.506)	0.326 (0.203‒0.834)	0.010 (1.000)	0.445 (0.000)	NC
	II	−0.105 (0.440)	1.920 (0.873)	11.896 (0.012‒97.896)	0.292 (0.110)	0.106 (0.120)	NC
	III	−0.599 (0.306)	−21.123 (0.004)	2.605 (0.227‒9.494)	0.012 (0.770)	0.009 (0.390)	0.060 (0.005‒0.220)

NC, not calculated; *SSD*, sum of squared deviations; *H*_Rag_, Harpending’s raggedness index.

*t*_min_: time estimated basing on the minimum cpDNA substitution rate (1 × 10^−9^ s s^−1^y^−1^).

*t*_max_: time estimated basing on the maximum cpDNA substitution rate (3 × 10^−9^ s s^−1^y^−1^).

### Present and past distribution modelling

**Figure 5:**
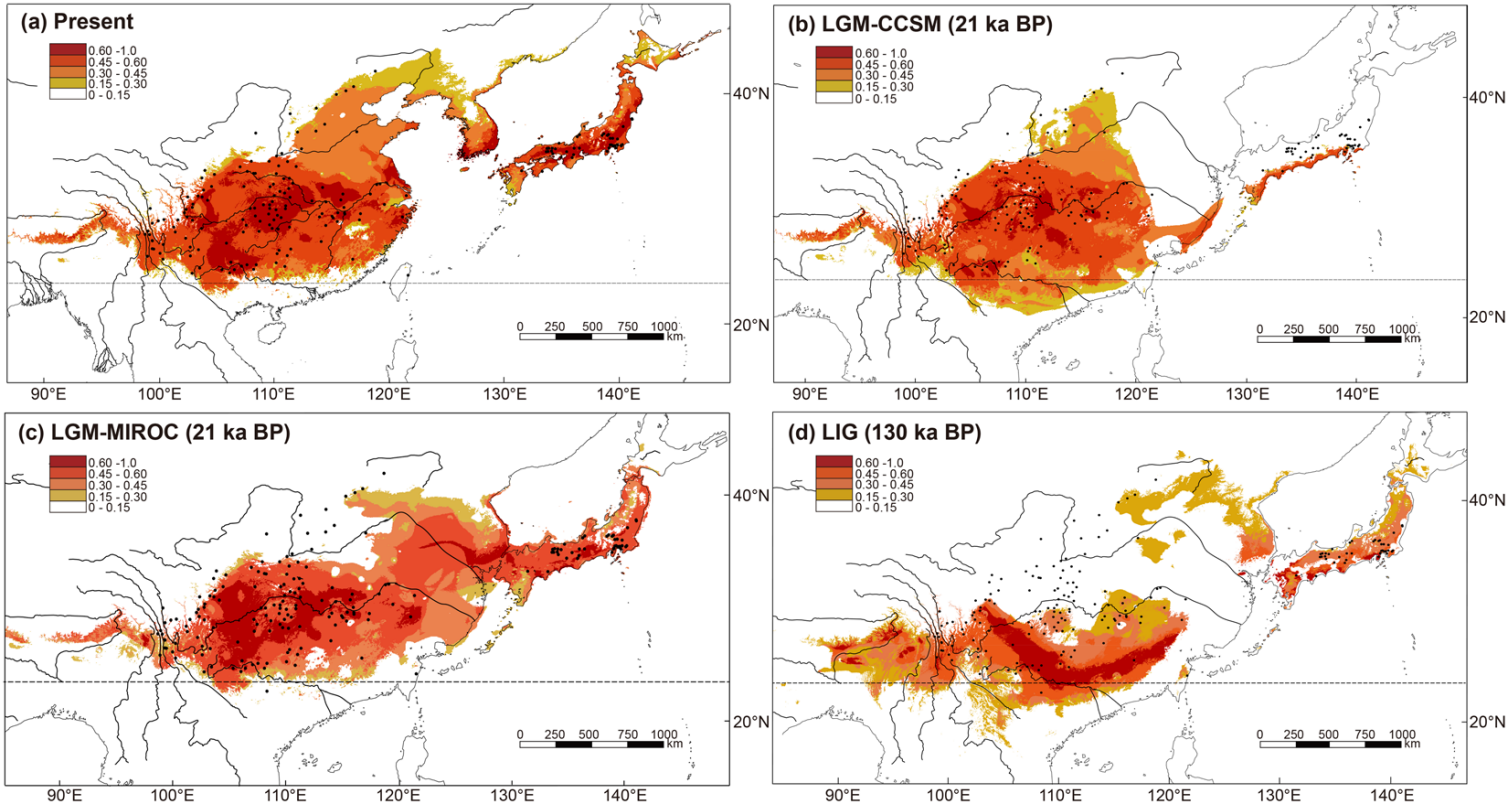
Modelled climatically suitable areas for *Indigofera bungeana* complex at different times using Maxent. Niche model results were modified in ArcGIS version 10.0 (http://www.esri.com/software/arcgis/arcgis-for-desktop). (**a**) The present; the last glacial maximum (LGM: c. 21 ka BP) under the (**b**) CCSM and (**c**) MIROC models, and (**d**) the last interglacial (LIG: c. 130 ka BP). The logistic value of habit suitability is shown according to the color-scale bars.

The AUC value for the current potential distribution of the *I. bungeana* complex was high (0.984), indicating a good predictive model performance. The projection of the model over present bioclimatic conditions shows a good habit suitability between 23°N and 45°N in East Asia (Fig. 5a). With 0.15 chosen as the threshold suitability, the CCSM (Fig. 5b) and MIROC (Fig. 5c) models yielded largely similar paleo-distributions in the LGM, while the MIROC inferred a more similar distribution range to the present day. However, the areas with high suitability (>0.60) were slightly decreased but significantly fragmented in both the CCSM and MIROC models compared with the present distribution, indicating possible habitat loss and fragmentation during the LGM.

## Discussion

### Pleistocene expansion

Chloroplast and nuclear datasets revealed high levels of genetic diversity of *I. bungeana* complex (Table 4). In addition, genetic differentiation among ‘the assumed species’ were very small, but extremely high among populations or regions using either cpDNA or nuclear DNA markers (Table 4). The small genetic differentiations seem not to support the previous taxonomic delimitations within this complex^17^. Relatively few haplotypes but high proportions of private haplotypes were detected across the investigated populations or regions in this study (Table 1). Most populations across the different regions are dominated by numerous private haplotypes although a few common haplotypes were also found between populations of the local regions (Figs 3 and 4).

This phylogeographic pattern may be better explained by the hypothesis that all examined populations have experienced a common expansion followed by the fast isolations^29^. The star phylogeny of haplotypes, an evidence of common expansion, was also observed for both chloroplast and nuclear haplotypes (Figs 3 and 4). The BSP analysis, which is based on coalescent methods, revealed that the effective population sizes (*N*_e_) increased in the early and middle Pleistocene. What’s more, the results of mismatch distribution and neutral tests further supported the expansion hypothesis. All these available evidences seem to support that a common expansion have occurred within *I. bungeana* complex. The range expansions detected in cpDNA clade C and nuclear clade III were estimated to have occurred approximately between 60 and 961 Kya, in the early and middle Pleistocene. Although we could not pinpoint the expansion accurately, it is highly possible that climate change of the Quaternary might have facilitated this expansion^30^. Some cold-tolerant plants, such as the species of the fir genus, expanded extensively and continuously in high-elevation regions during the Quaternary^31–34^.

However, it remains unknown how the ancestral haplotypes disappeared in different regions/populations. It is highly likely that geographic isolations following the range expansions promoted the private haplotypes displaced the ancestral ones. Such scenarios were usually found for isolated species or populations^35–37^. The Quaternary climate changes after the range expansions should have mainly accounted for such fixtures of the private haplotypes in the different regions and great among-regions differentiations. The limited seed and pollen dispersals of this specie complex may have also played an important role.

### *In situ* survival in most distributions of the complex during the LGM

Although northern and southern China have never been covered by large ice sheets during the LGM, it is estimated that the climate was cooler by at least 7‒10/4‒6 °C and dryer by c. 200‒300/400‒600 mm yr^−1^ than the present, respectively^38–40^. The Quaternary climate changes have strongly affected distribution and genetic diversity of the temperate plants in East Asia, resulted in experienced glacial southward migrations, or *in situ* glacial survival^18, 26^. Species of the *I. bungeana* complex have high cold tolerance as well as drought tolerance with the most northward distributions between c. 23°N and 45°N in the genus^41^.

Most private haplotypes in this complex derived from the range expansion in the early and middle Pleistocene, earlier than the LGM. Our simulations of the distributions of the *I. bungeana* complex during the LGM also indicated that the distribution did not migrate southward significantly although the core distributions shrank, which is also consistent with the species with similar distribution in East Asia, such as *Juglans cathayensis* ^42^, *Quercus variabilis* ^43^, and *Tetrastigma hemsleyanum* ^44^.

Given the above evidence, we tentatively suggested that the *I. bungeana* complex might have survived *in situ* or in multiple large refugia in response to the climate change of the LGM. However, this and other Quaternary climate oscillations might have together accelerated the regional isolations of the *I. bungeana* complex that promoted the fixture of the numerous private haplotypes. Our results seem to suggest not all woody species growing under the temperate deciduous forests in northern China migrated southward^3^. However, these climatic changes might have promoted the species or genetic differentiations of plants occurring in East Asia as suggested by Qian and Ricklefs^2^.

## Methods

### Population sampling

A total of 472 individuals representing four species were collected from 51 populations, with 1‒13 individuals per population (spaced at least 50 m apart), covering the whole range of the *I*. *bungeana* complex in China (see Supplementary Table S2). We tentatively ascribed all collected materials to four species names in the Flora of China^17^. Voucher specimens are deposited in Herbarium of Chengdu Institute of Biology, Chinese Academy of Sciences (CDBI).

### DNA extraction, amplification and sequencing

Total genomic DNA was extracted from silica-gel-dried leaves using the plant genomic DNA extraction kit (TIANGEN Biotech., Beijing, China). After preliminary screening of ten chloroplast fragments (i.e., *atp*F-*atp*H, *rpl*32-*trn*L, *rps*16-*trn*Q, *ndh*J-*trn*F, *mat*K, *trn*L-*trn*F, *ndh*C-*trn*V, *trn*D-*trn*T, *ndh*F-*rpl*32 and *psb*A-*trn*H) for the representative samples of *I*. *bungeana* complex, we chose two cpDNA intergenic spacer (IGS) regions (*ndh*J-*trn*F and *trn*D-*trn*T)^45–47^ for the phylogeographic study. In addition, *Pgk1*, a single-copy nuclear (scn) gene responsible for coding plastid phosphoglycerate kinase isoenzymes, was surveyed among 51 populations using two new primers (see Supplementary Table S3) designed on the basis of sequences obtained using the primers of Huang *et al*.^48^. All ﻿the chlorotypes and nuclear haplotypes from *Indigofera bungeana* were deposited in GenBank under accession numbers (submitted).

All amplifications were performed in 25 µL reactions containing 17 µL deionized sterile water, 1.5 µL of 25 mM MgCl_2_, 2.5 µL Taq reaction buffer, 2 µL of 2.5 mM dNTP, 0.5 µL of each primer at 10 pmol mL^−1^, 0.5 µL (2.5 unit) Taq DNA polymerase (TIANGEN, Beijing, China), and 0.5 µL genomic DNA (10‒50 ng). The PCR amplifications were performed as follows: initial denaturation at 94 °C for 5 min, followed by 33 cycles of denaturation at 94 °C for 45 s, annealing (54 °C, 30 s for *ndh*J-*trn*F and *trn*D-*trn*T; 58 °C, 30 s for *Pgk1*), and extension at 72 °C (1 min for *ndh*J-*trn*F; 90 s for *trn*D-*trn*T; 45 s for *Pgk1*), and a final extension at 72 °C for 7 min prior to holding at 12 °C forever. PCR products were purified using an E.Z.N.A gel extraction kit (OMEGA, Biotech., USA). The purified PCR products were sequenced by Life Technologies^TM^ (Shanghai, China).

### Population genetic analyses

Sequences were assembled and edited with Sequencher 4.1 (Gene Codes, Ann Arbor, MI), aligned using Clustal X 1.81^49^ and subsequent manual adjustments. Nuclear (*Pgk1*) allelic phases were resolved using the algorithm of PHASE^27^ implemented in DnaSP 5.0^50^, using 1,000 iterations with a 1,000 generation burn-in iterations and a thinning interval of 10. Indels were treated as single mutation events and coded as substitution (A or T). Haplotypes of cpDNA (chlorotypes) and *Pgk1* were recognized using DNASP 5.0^50^. Genealogical relationships among chlorotypes and nuclear (*Pgk1*) haplotypes were constructed using a statistical parsimony algorithm^51^ as implemented in Network v. 4.6 (http://fluxus-engineering.com).

Population gene diversity (*H*_S_, *H*_T_) and between-population differentiation (*G*_ST_, *N*_ST_) were estimated using PERMUT^52^ with the 1,000 permutations test. A higher *N*_ST_ than *G*_ST_ usually indicates the presence of phylogeographic structure, that is, the more frequent occurrence of closely related haplotypes in the same area than less closely related haplotypes^52^. A comparison was made between *N*_ST_ and *G*_ST_ using the *U*-statistics test.

The spatial analysis of molecular variance (SAMOVA) was conducted using SAMOVA 1.0^53^ to define the groups of populations that are geographically homogeneous and maximally differentiated. The SAMOVA analysis was conducted with the number of groups (*K*) ranging from 2 to 20. To verify the consistency, we ran the analysis five times for each *K* value with 1,000 independent iterations, starting from 100 random initial conditions. We assessed the optimal *K* as the one for which *F*_CT_ (i.e., the genetic variance owing to divergences between groups) was the highest and significant.

Hierarchical analysis of molecular variance (AMOVA) was performed in ARLEQUIN 3.1^54^ to estimate the partition of genetic variance among groups, within and among populations. In the AMOVA analysis, populations were partitioned by geography or species, respectively. Geographical groups were obtained from SAMOVA analysis. If the SAMOVA analysis was unable to detect suitable groups, populations were grouped following Wu & Wu^28^.

### Phylogenetic analyses and divergence time estimation

Two species of *Indigofera* (*I*. *szechuensis* Craib and *I*. *lenticellata* Craib) were chosen as outgroups in the phylogenetic analyses according to the phylogenetic analyses of *Indigofera* (our unpublished results). Phylogenetic relationships of the chlorotypes and nuclear (*Pgk1*) haplotypes were reconstructed with Neighbor-joining (NJ) and Bayesian inference (BI) methods, using MEGA 5.05^55^ and MrBayes 3.1^56^, respectively. In the NJ analysis, we used the Kimura’s 2-parameter model^57^. Confidence values at the nodes were tested by performing 1,000 bootstrap replicates. Prior to BI analyses, the optimal nucleotide substitution model was determined using jModeltest 2.1.2^58^ via the Akaike Information Criterion (AIC)^59^. The TVM + I + G model (cpDNA) and GTR + I + G model (*Pgk1*) were selected as the best-fit models. Four Markov chain Monte Carlo (MCMC) chains were run for 20,000,000 generations, starting from random trees and sampling one tree per 1,000 generations with the first 4,000,000 samples discarded as burn-in. The program Tracer 1.5^60^ was used to check the parameter convergence and effective sample size. A 50% majority-rule consensus tree was summarized with posterior probabilities as nodal support.

A likelihood-ratio test^61^ in PAUP 4.10b^62^ suggested that the chloroplast and nuclear datasets rejected a strict molecular clock (*P* < 0.01), therefore we used a relaxed molecular clock. Divergence times between the chloroplast and nuclear haplotype clades were estimated under a Bayesian approach^63^ in BEAST 1.6.2^64^. As there is no fossil record of *Indigofera*, we adopted a substitution rate method. The cpDNA substitution rates for most angiosperm species have been estimated to vary between 1.0 × 10^−9^ and 3.0 × 10^−9^ substitutions per site per year (s s^−1^y^−1^)^65^. Given the uncertainties of these rate values in *Indigofera*, we used a minimum (1.0 × 10^−9^ s s^−1^y^−1^), mean (2.0 × 10^−9^ s s^−1^y^−1^) and maximum (3.0 × 10^−9^ s s^−1^y^−1^) substitution rate, respectively. For *Pgk1*, we adopted a nucleotide substitution rate of 13.6 × 10^−9^ s s^−1^y^−1^ according to Huang *et al*.^66^.

### Demographic analyses

To test the assumption of selective neutrality, we performed Tajima’s *D* ^67^ and Fu’s *F*_S_^68^ tests, which are expected to show significant negative values under population expansion and positive under a population bottleneck. A mismatch distribution analysis^69^ (MDA) was also conducted to explore the demographic history of major chlorotypes and nuclear (*Pgk1*) haplotype clades. Populations that have experienced expansion are expected to have a unimodal shape, whereas stable populations are expected to have a bi- or multi-modal mismatch distribution. The goodness-of-fit was assessed by the sum of squared deviations (*SSD*), Harpending’s raggedness index^70^ (*H*_Rag_) and 95% confidence interval (CI) around *τ* under a sudden-expansion model. Statistical significance was determined by 1,000 bootstrap replicates. These analyses were conducted using ARLEQUIN 3.1^58^. To obtain estimates of changes in demographic growth over the history of major clades, the historical demographic dynamics of the *I. bungeana* complex were inferred from Bayesian skyline plot (BSP) analyses using BEAST 1.6.2^64^. Linear and stepwise models were explored using an uncorrelated lognormal relaxed clock. Runs consisted of 50,000,000 generations, with trees sampled every 1000 generations. The BSP was visualized in the program Tracer version 1.5, which summarizes the posterior distribution of population size over time.

### Present and past distribution modelling

We used the maximum entropy modelling implemented in MAXENT 3.3.3k^71^ to infer the potential geographic range of the *I. bungeana* complex at the present, the LGM (ca. 21 ka) and last interglacial (LIG, ca. 130 ka) based on the bioclimatic layers downloaded from the WorldClim database^72^ (http://www.worldclim.org) at 2.5-arcmin resolution. The paleo-climatic conditions during the LIG were simulated by Community Climate System Model^73^ (CCSM), while we used two available models for the LGM: CCSM and MIROC^74^. Distribution records of the species of *I. bungeana* complex were sourced from the database of GBIF (http://www.gbif.org/) and Chinese Virtual Herbarium (http://www.cvh.org.cn), as well as our own field collections. After initial screening for duplicates and records aggregation into a 2.5 resolution raster, 181 unique records were used. Highly correlated variables (r > 0.7) were excluded, and we ultimately selected five bioclimatic variables (i.e., annual mean temperature, temperature seasonality, mean temperature of driest quarter, precipitation of wettest month, and precipitation of warmest quarter). To statistically evaluate model performance, we used the area under the “Receiver Operating Characteristic (ROC) Curve”^75^ (AUC), a threshold-independent measure of model performance as compared to null expectations.

## Electronic supplementary material

Supplementary Information
