# Supplementary material for: The demographic response of a deciduous shrub (the Indigofera bungeana complex, Fabaceae) to the Pleistocene climate changes in East Asia
Source: Sci Rep. 2017 Apr 6;7:697. doi: 10.1038/s41598-017-00613-x (PMC5428846; doi:10.1038/s41598-017-00613-x)
Supplement: Supplementary file 1 — Supplementary Information [file 41598_2017_613_MOESM1_ESM.pdf]

The demographic response of a deciduous shrub (the *Indigofera bungeana* complex, Fabaceae) to the Pleistocene climate changes in East Asia

Xue-Li Zhao[1,2], Xin-Fen Gao[1], Zhang-Ming Zhu[3], Yun-Dong Gao[1] and Bo Xu[1]

[1]*CAS Key Laboratory of Mountain Ecological Restoration and Bioresource Utilization & Ecological Restoration and Biodiversity Conservation Key Laboratory of Sichuan Province, Chengdu Institute of Biology, Chinese Academy of Sciences, P.O. Box 416, Chengdu, Sichuan 610041, China,* [2]*College of Forestry, Southwest Forestry University, Kunming 650224, China,*

[3]*Institute of Ecology and Geobotany, Yunnan University, Kunming 650091, China*.

Correspondence and requests for materials should be addressed to X.-F.G. (e-mail: xfgao@cib.ac.cn)

Supplementary Information

Supplementary Figure S1. Correlation between the number of groups (*K*) and genetic variance (F_{CT}) in SAMOVA analysis.

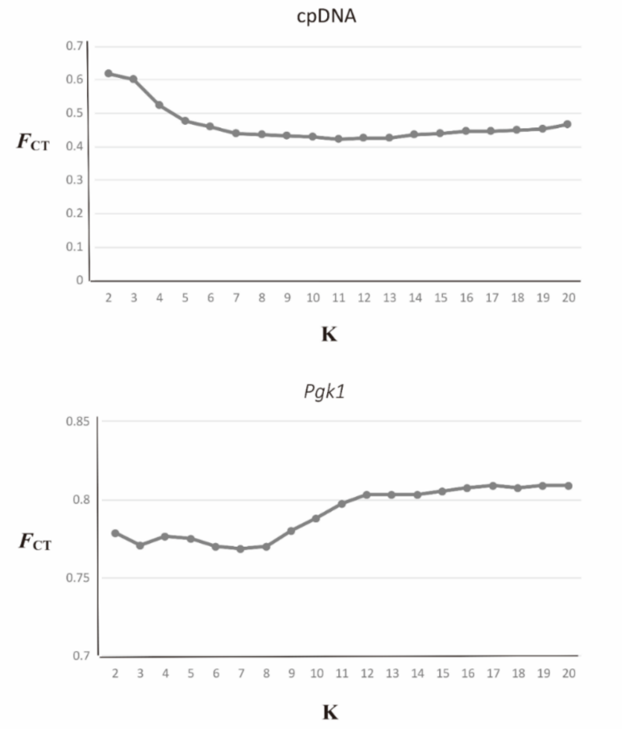

Supplementary Figure S2. Maximum parsimony network of 133 cpDNA haplotypes identified from areas.

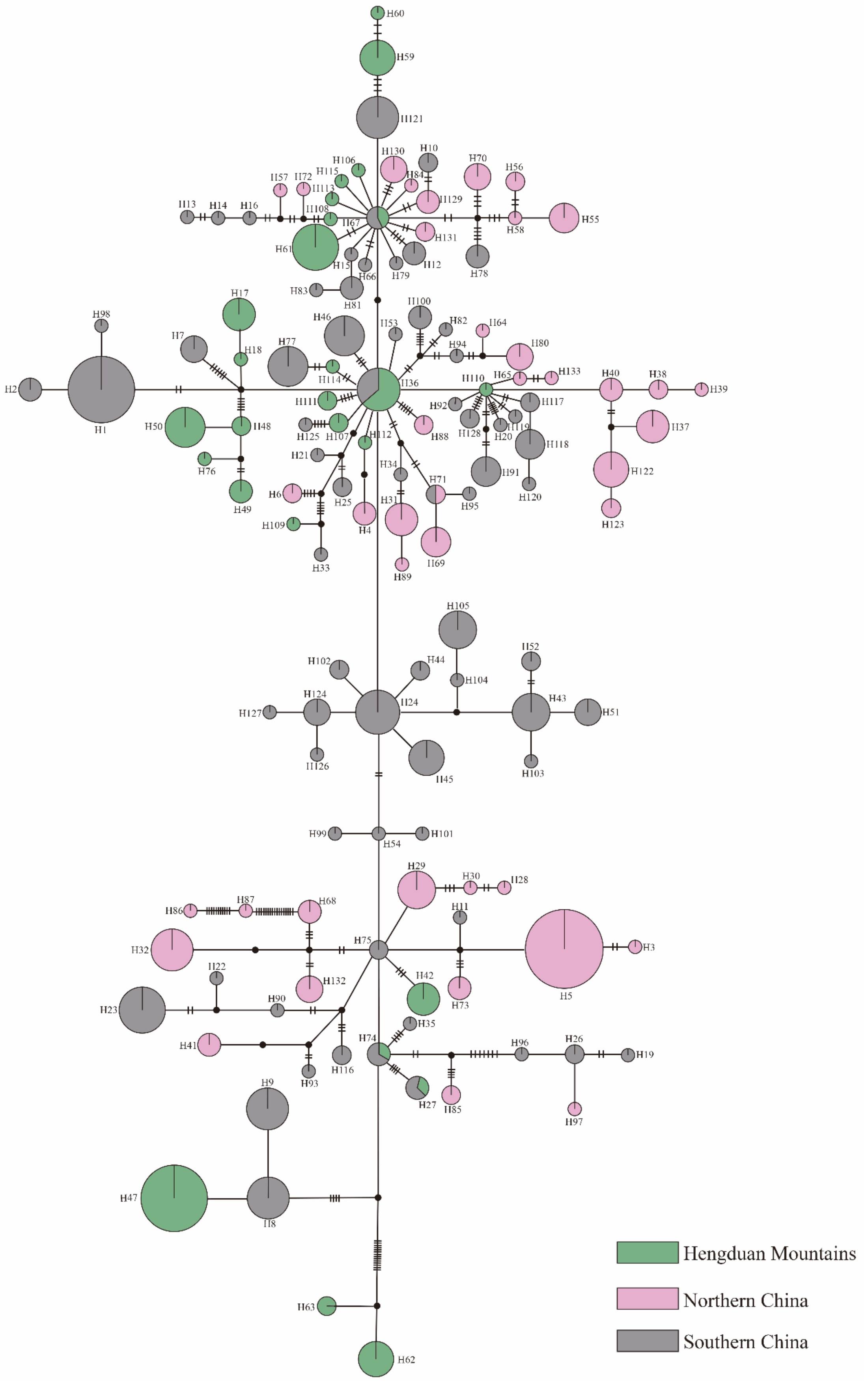

Supplementary Figure S3. Maximum parsimony network of 68 *Pgk1* haplotypes identified from areas.

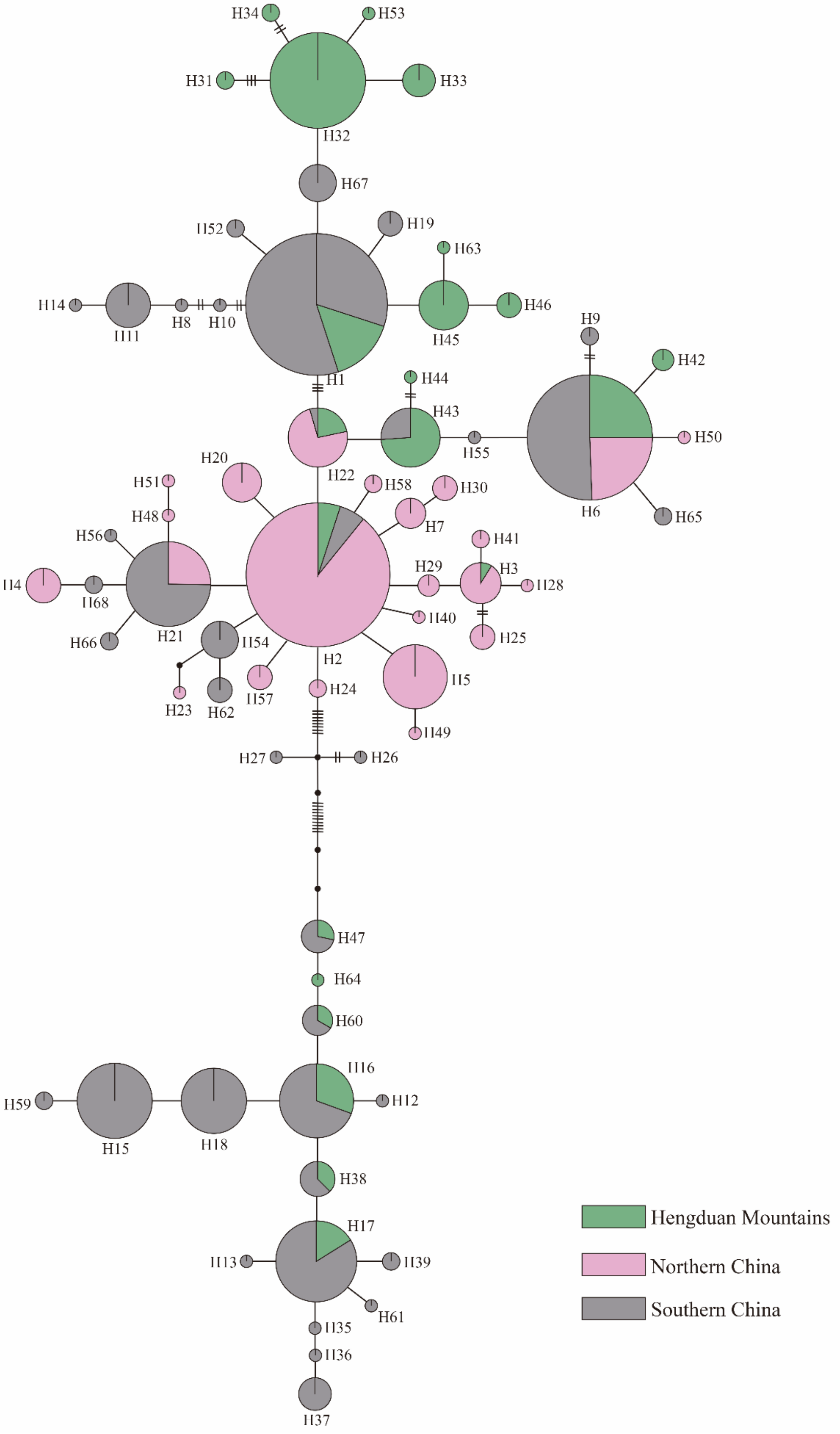

Supplementary Figure S4. Maximum parsimony network of 133 cpDNA haplotypes identified from species.

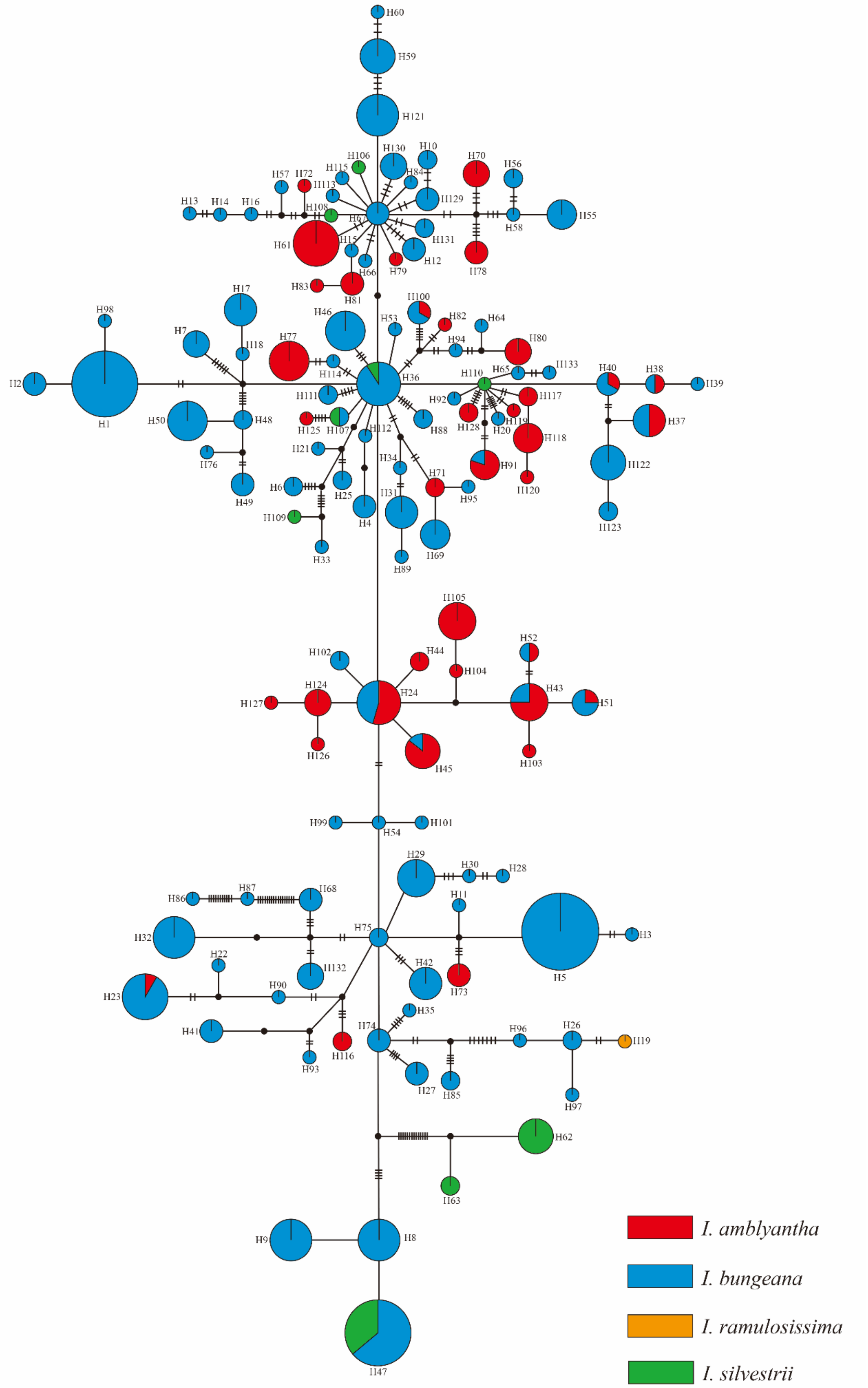

Supplementary Figure S5. Maximum parsimony network of 68 *Pgk1* haplotypes identified from species.

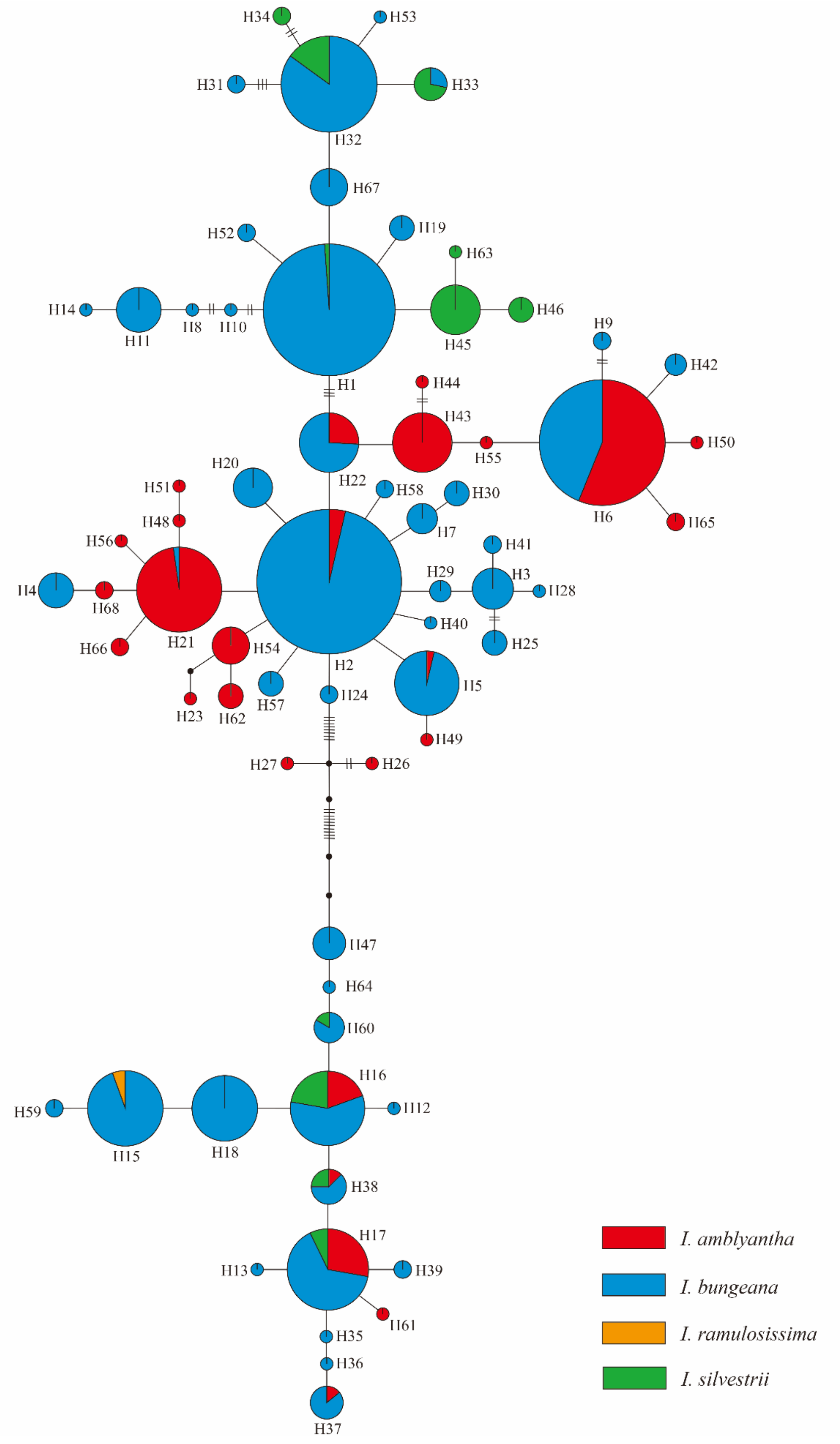

Supplementary Figure S6. Mismatch distribution analysis for cpDNA and *Pgk1* sequence data.

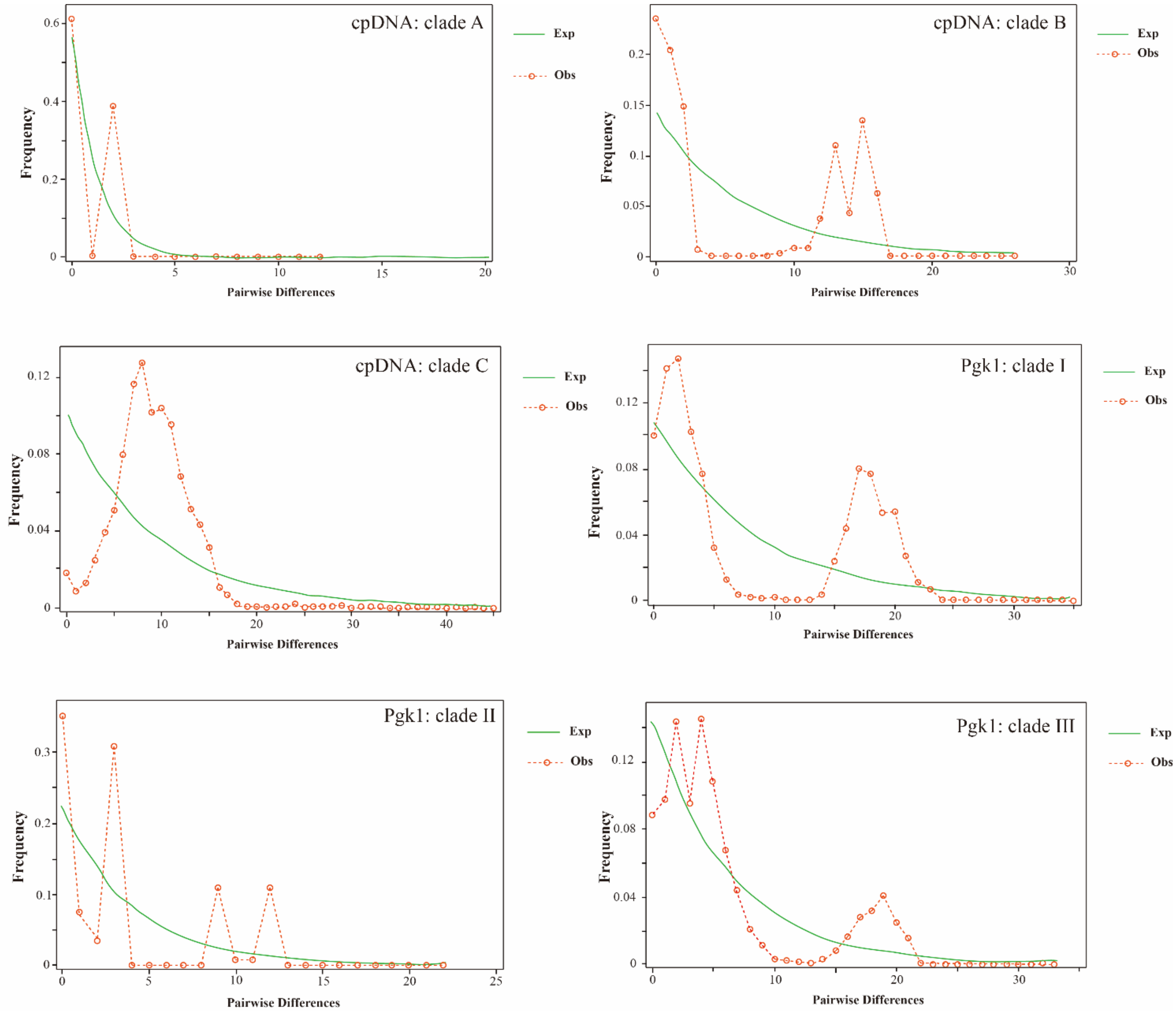

Supplementary Figure S7. Bayesian skyline plot for the same clades as in mismatch, showing the effective population size fluctuation throughout time. X axis: no. of substitutions, Y axis: is Ne*µ (effective population size * substitution rate in generation). The middle line is the median estimate, upper and lower limits (95% HPD) are also given.

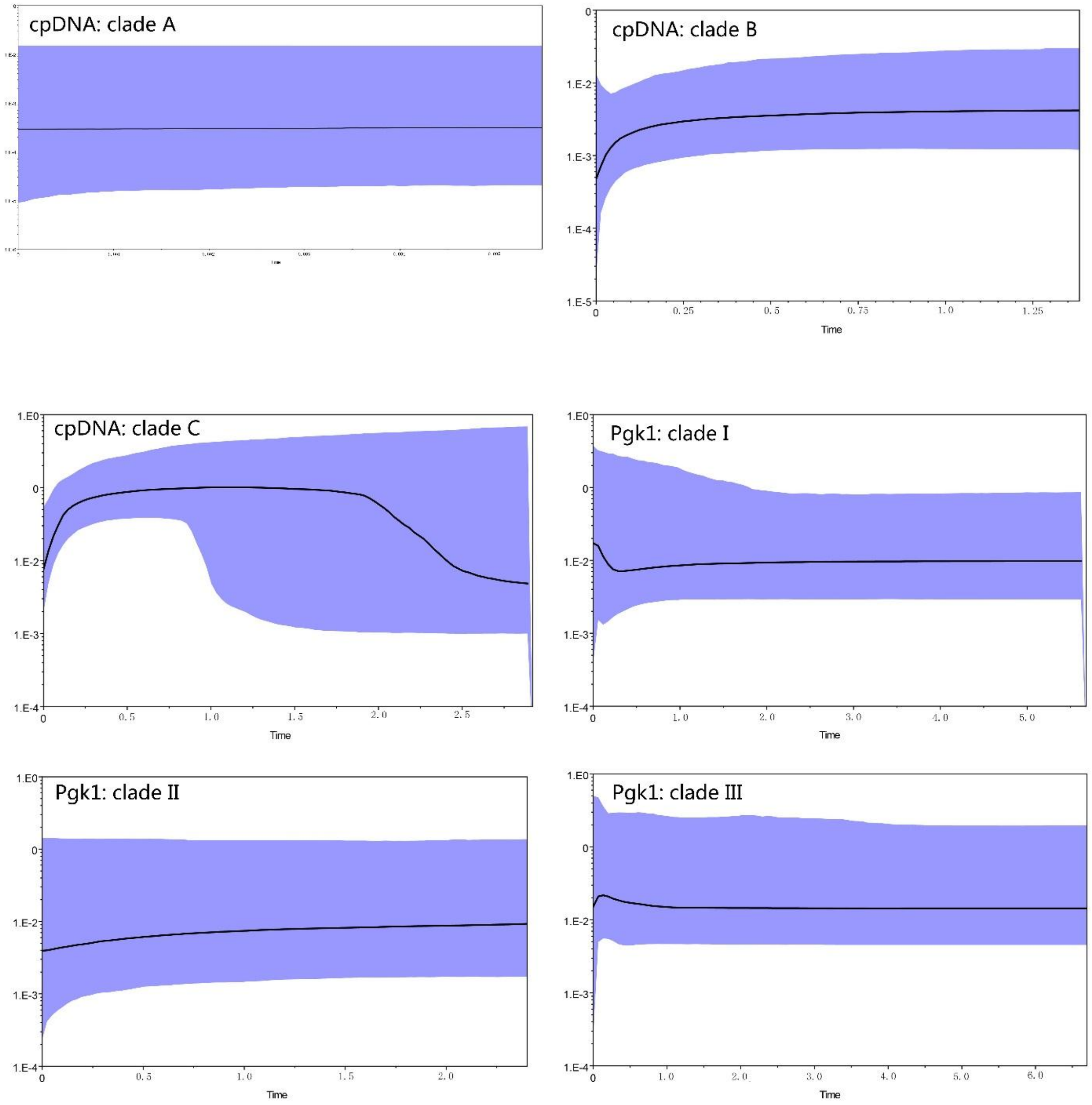

Supplementary Table S1. Divergence times (Ma, million years ago) of major cpDNA and Pgk1 nodes (Figs. 3a & 4a) estimated by BEAST analyses. Time 1, 2 and 3 represent divergence times estimated using a cpDNA substitution rate of 1×10^{-9} s s^{-1}y^{-1}, 2×10^{-9} s s^{-1} y^{-1} and 3×10^{-9} s s^{-1} y^{-1}, respectively.

cpDNA					*Pgk1*
nodes	Time 1 (95% HPD)	Time 2 (95% HPD)	Time 3 (95% HPD)	nodes	Time (95% HPD)
a1	3.69 (2.65–4.77)	1.82 (1.3–2.31)	1.3 (0.88–1.62)	b1	1.47 (0.75–2.37)
a2	0.51 (0–1.73)	0.27 (0–0.78)	0.24 (0–0.52)	b2	0.67 (0.26–1.28)
a3	4.87 (3.38–6.39)	2.44 (1.65–3.23)	1.73 (1.17–2.24)	b3	1.15 (0.62–1.79)
a4	0.62 (0.02–1.55)	0.31 (0.02–0.77)	0.27 (0.01–0.5)	b4	0.35 (0.01–0.93)
a5	3.36 (2.43–4.25)	1.65 (1.21–2.1)	1.19 (0.88–1.62)	b5	0.98 (0.51–1.66)

Supplementary Table S2. Voucher information of populations of *Indigofera bungeana* complex sampled in this study. CDBI = Herbarium of Chengdu Institute of Biology, Chinese Academy of Sciences.

Species	Population code	Location	Voucher
I. amblyantha	JY1	Jiyuan, Henan	*X.L. Zhao* & *C.Q. Peng* 195 (CDBI)
	JZ	Jinzhai, Anhui	*X.L. Zhao* & *C.Q. Peng* 59 (CDBI)
	MX2	Maoxian, Sichuan	*X.F. Gao* & *X.L. Zhao* 15878 (CDBI)
	RC	Ruicheng, Shannxi	*X.L. Zhao* & *C.Q. Peng* 192 (CDBI)
	SNJ	Shennongjia, Hubei	*X.L. Zhao* & *C.Q. Peng* 263 (CDBI)
	SQ	Shiquan, Shannxi	*X.L. Zhao* & *C.Q. Peng* 208 (CDBI)
	TMS2	Tianmushan, Zhejiang	*X.L. Zhao* & *C.Q. Peng* 95 (CDBI)
	WN2	Wuning, Jiangxi	*X.L. Zhao* & *C.Q. Peng* 105 (CDBI)
	WN3	Wuning, Jiangxi	*X.L. Zhao* & *C.Q. Peng* 106 (CDBI)
	XIX1	Xixia, Henan	*X.L. Zhao* & *C.Q. Peng* 198 (CDBI)
	XY	Xinyang, Henan	*X.L. Zhao* & *C.Q. Peng* 36 (CDBI)
	AL	Anlong, Guizhou	*Z.M. Zhu* & *W.B. Ju* 379 (CDBI)
	CP	Changping, Beijing	*X.L. Zhao* & *C.Q. Peng* 162 (CDBI)
	DFA	Dafang, Guizhou	*Z.M. Zhu* & *W.B. Ju* 312 (CDBI)
	DFE	Danfeng, Shannxi	*X.L. Zhao* & *C.Q. Peng* 203 (CDBI)
	DQ	Deqin, Yunnan	*X.L. Zhao* et al. 2014-87 (CDBI)
I. bungeana	HS	Huangshan, Anhui	*X.L. Zhao* & *C.Q. Peng* 97 (CDBI)
	HZ	Huozhou, Shanxi	*X.L. Zhao* & *C.Q. Peng* 191 (CDBI)
	JN	Jinan, Shandong	*X.L. Zhao* & *C.Q. Peng* 186 (CDBI)
	JR	Jurong, Jiangsu	*X.L. Zhao* & *C.Q. Peng* 63 (CDBI)
	JY2	Jiyuan, Henan	*X.L. Zhao* & *C.Q. Peng* 196 (CDBI)
	JZG	Jiuzhaigou, Sichuan	*X.F. Gao* 1 (CDBI)
	KLQQ	Kelaqinqi, Neimenggu	*X.L. Zhao* & *C.Q. Peng* 173 (CDBI)
	LC	Liangcheng, Fujian	*X.L. Zhao* & *C.Q. Peng* 114 (CDBI)
	LD1	Luding, Sichuan	*X.F. Gao* & *X.L. Zhao* 15913 (CDBI)
	LD3	Luding, Sichuan	*X.F. Gao* et al. 9360 (CDBI)
	LS	Lushan, Jiangxi	*X.L. Zhao* & *C.Q. Peng* 103 (CDBI)
	LY	Leye, Guangxi	*Z.M. Zhu* & *W.B. Ju* 422 (CDBI)
	MEX	Meixian, Shannxi	*X.L. Zhao* & *C.Q. Peng* 207 (CDBI)
	MX1	Maoxian, Sichuan	*X.F. Gao* & *X.L. Zhao* 15868 (CDBI)

	MY	Miyun, Beijing	*X.L. Zhao* & *C.Q. Peng* 165 (CDBI)
	NC	Nanchuan, Chongqin	*Y.H. Tong* 0913 (CDBI)
	NQ	Neiqiu, Hebei	*X.L. Zhao* & *C.Q. Peng* 189 (CDBI)
	SME	Shimen, Hunan	*Z.M. Zhu* & *W.B. Ju* 614 (CDBI)
	SMI	Shimian, Sichuan	*X.L. Zhao* et al. 2014-142 (CDBI)
	SX	Shexian, Hebei	*X.L. Zhao* & *C.Q. Peng* 190 (CDBI)
	TMS1	Tianmushan, Zhejiang	*X.L. Zhao* & *C.Q. Peng* 94 (CDBI)
	TT	Tiantai, Zhejiang	*X.L. Zhao* & *C.Q. Peng* 84 (CDBI)
	WM	Wangmo, Guizhou	*Z.M. Zhu* & *W.B. Ju* 400 (CDBI)
	WN1	Wuning, Jiangxi	*X.L. Zhao* & *C.Q. Peng* 104 (CDBI)
	WT	Wutai, Shanxi	*X.L. Zhao* & *C.Q. Peng* 135 (CDBI)
	WX2	Weixi, Yunnan	*X.L. Zhao* et al. 2014-74 (CDBI)
	XIX2	Xixia, Henan	*X.L. Zhao* & *C.Q. Peng* 202 (CDBI)
	XX	Xingxian, Shanxi	*X.L. Zhao* & *C.Q. Peng* 130 (CDBI)
	YJ	Yinjiang, Guizhou	*Z.M. Zhu* & *W.B. Ju* 734 (CDBI)
	ZD	Zhidan, Shannxi	*X.L. Zhao* & *C.Q. Peng* 126 (CDBI)
	ZL	Zhuolu, Hebei	*X.L. Zhao* & *C.Q. Peng* 161 (CDBI)
I. ramulosissima	HL	Hualian, Taiwan	*X.F. Gao* & *Y. Zhang* 15779 (CDBI)
I. silvestrii	LD2	Luding, Sichuan	*X.F. Gao* et al. 9322 (CDBI)
	MX3	Maoxian, Sichuan	*X.F. Gao* & *X.L. Zhao* 15893 (CDBI)
	WX1	Weixi, Yunnan	*X.L. Zhao* et al. 2014-71 (CDBI)

Supplementary Table S3. The primers used for amplification and sequencing in this study.

Region	Primer	Primer sequence (5'–3')	Source
*ndh*J-*trn*F	ndhJ	ATGCCYGAAAGTTGGATAGG	Shaw *et al.*[45]
	TabE	GGTTCAAGTCCCTCTATCCC	Taberlet *et al.*[43]
*trn*D-*trn*T	trnD-F	ACCAATTGAACTACAATC CC	Demesure *et al.*[44]
	trnT	CTACCACTGAGTTAAAAGGG	Demesure *et al.*[44]
Pgk1	PGK-CF	TTAGCATCAGCAGCAAAC	This study
	PGK-CR	GTTGAGAAGTTGGTGGC	This study
